# Identifying Sequence Variants of 18 Hereditary Ovarian Cancer-Associated Genes in Chinese Epithelial Ovarian Cancer Patients

**DOI:** 10.1155/2021/5579543

**Published:** 2021-07-24

**Authors:** Xiao Wu, Zhengzheng Chen, Pingping Ren, Xuxu Zhao, Dongdong Tang, Hao Geng, Xiaofeng Xu, Weidong Zhao

**Affiliations:** ^1^Reproductive Medicine Center, Department of Obstetrics and Gynecology, The First Affiliated Hospital of Anhui Medical University, Hefei, Anhui, China; ^2^Anhui Province Key Laboratory of Reproductive Health and Genetics, Hefei, Anhui, China; ^3^Biopreservation and Artificial Organs, Anhui Provincial Engineering Research Center, Anhui Medical University, Hefei, Anhui, China; ^4^Department of Obstetrics and Gynecology, Anhui Provincial Hospital affiliated to Anhui Medical University, Hefei, Anhui, China; ^5^Department of Obstetrics and Gynecology, The First Affiliated Hospital of University of Science and Technology of China, Hefei, Anhui, China

## Abstract

**Objectives:**

The causes of ovarian cancer (OC) have been confirmed to be closely related to genetic factors. Identifying sequence variants of hereditary ovarian cancer (HOC) susceptibility genes can increase clinical surveillance, facilitate early detection, and provide personalized treatment for patients. This study is aimed at investigating the variation frequency of HOC susceptibility genes in the Chinese population and providing information for the etiology and genetics of OC.

**Methods:**

118 epithelial OC patients were recruited in this clinical study. Variants of 18-gene panel were detected in blood samples by next-generation sequencing (NGS) technology.

**Results:**

Overall, 36.44% (43/118) of patients carried at least one pathogenic variant. Among these, *BRCA1* pathogenic variants were detected in 31 (26.27%) patients, and 5 (4.24%) patients carried pathogenic variants of *BRCA2*. Moreover, 27.12% (32/118) of patients carried variants of unknown significance (VUSs). Importantly, we detected eight variants that were not reported previously.

**Conclusions:**

Our study enlarged the spectrum of HOC-associated gene sequence variants in the Chinese population and also proved the necessity of multigene testing in epithelial OC patients. The identification of patients with HOC will allow family members to undergo cascade testing where identification of unaffected carriers can facilitate early detection, risk reduction, or prevention of OC and ultimately improve long-term outcomes.

## 1. Introduction

The average annual ovarian cancer (OC) incidence rate was 9.5 per 100,000 women in 2013–2017 in Asians [1]. The National Central Cancer Registry of China estimated that in 2015, approximately 52,100 people in China were newly diagnosed with OC, and 22,500 people died from the disease [2]. Due to the development of diagnostic capabilities, the number of newly diagnosed cases each year is increasing gradually. Epithelial cancers are most common among OC, accounting for 90% of all cases [3]. Based on tumor cell histology, epithelial carcinoma can be classified into serous (52%), endometrioid (10%), mucinous (6%), clear cell (6%), and unspecified subtypes. The 5% death rate makes it the fifth leading cause of cancer-related deaths in women [4]. Due to the lack of early specific symptoms, most OC patients are diagnosed at an advanced stage. More than half of the serous carcinomas are diagnosed at stage III, and nearly 30% are diagnosed at stage IV. The five-year cause-specific survival rates of these patients are 42% and 26%, respectively [3]. However, the five-year survival rate of OC diagnosed in the local stage is 93% [1]. The prevention and early detection of OC are challenges faced by gynecological oncologists.

Several studies have shown some high-risk factors related to OC, such as genetic factors, older menopausal age, obesity, menopausal hormone therapy use, a history of endometriosis, and smoking [5] Among these factors, genetic factors are the most definitive cause. Multiple studies have shown that women with germline pathogenic variants (PVs) of hereditary ovarian cancer- (HOC-) related genes have an increased risk of developing OC. Since Hall et al. [6] discovered *BRCA1* (MIM 113705) in 1990 and Wooster et al. [7] discovered *BRCA2* (MIM 600185) 4 years later, PVs of these two genes are known to be the leading cause of HOC. The lifetime risk of developing OC in general population is 1.3%, but for some specific women (*BRCA1*/*BRCA2* PVs carriers), it can be as high as 44% and 17%, respectively [3, 8]. Nevertheless, recent studies have identified more PVs of genes other than *BRCA1* and *BRCA2* [9].The risk of OC may also increase by PVs of Lynch syndrome-related DNA Mismatch repair (*MMR*) genes (*MSH2*, *MLH1*, *PMS2*, etc.), Li-Fraumeni syndrome-related tumor suppressant genes (*TP53*, etc.), and other genes in the homologous recombination repair mechanism (e.g., *ATM*, *CHEK2*, *RAD51*, *BRIP1*, and *PALB2*) [10].

The National Comprehensive Cancer Network (NCCN) guidelines clearly indicate that high-penetrance OC susceptibility gene testing should be provided for patients with epithelial OC diagnosed at any age [11]. The development of next-generation sequencing (NGS) technology makes it possible to analyze multiple cancer susceptibility genes simultaneously. This helps in saving costs, shortening the detection time, and providing unprecedented opportunities for molecular diagnosis of HOC. In addition, genetic testing can also help identify OC patients who are suitable to receive the poly-ADP ribose polymerase inhibitor treatment. There exist few studies on multigene panel testing of epithelial OC patients in the Chinese population. Hence, this study, using NGS technology to detect germline variants of the 18-gene panel in patients with epithelial OC, is aimed at investigating the variation frequency of HOC susceptibility genes in the Chinese population and providing information for the etiology and genetics of OC.

## 2. Materials and Methods

### 2.1. Study Cohort

The enrollment criteria for this study were patients with epithelial OC diagnosed at any age. From September 2016 to December 2018, clinicians continuously provided genetic testing for all patients diagnosed with epithelial OC in Anhui Provincial Cancer Hospital. All participants signed informed consent forms. Genetic counseling experts conducted information collection and genetic counseling to inform them of the content, methods, necessity, risks, and limitations of testing. This research project was approved by the Medical Ethics Committee of Anhui Provincial Cancer Hospital.

### 2.2. Sample Collection and NGS

5 milliliters of peripheral blood was collected from the study subjects, and EDTA was used as an anticoagulant. In accordance with the NCCN guidelines and published research articles, 18 cancer susceptibility genes (*BRCA1*, *BRCA2*, *CHEK2*, *PALB2*, *BRIP1*, *TP53*, *PTEN*, *STK11*, *CDH1*, *ATM*, *BARD1*, *MLH1*, *MSH2*, *MSH6*, *MUTYH*, *NBN*, *PMS2*, and *RAD51C*) (Table S1) were included in this panel for their possible role in the development of OC. The detection range included exon and adjacent ±10 bp intron regions (including point, deletion, and insertion variation). In addition, we used quantitative real-time polymerase chain reaction (qPCR) to validate copy number variants detected by target sequencing.

### 2.3. Germline Variants Classification

For PVs or likely PVs, verified by Sanger sequencing or qPCR (depending on the circumstances), the naming of variants were according to the rules recommended by the Human Genome Variation Society (http://www.hgvs.org/mutnomen/). Variants were classified into the following 5 categories according to the American College of Medical Genetics recommendations: class 1, benign (B); class 2, likely benign (LB); class 3, variant of uncertain significance(VUS); class 4, likely pathogenic (LP); and class 5, pathogenic (P) [12].

## 3. Results

### 3.1. Patient Characteristics

A total of 118 patients with epithelial OC were included in the study. The clinical characteristics and personal and family cancer history of the patients are displayed in Table 1. Age at diagnosis ranged from 31 to 79 years with an average age of 52 (±9.1) years. Serous cancer accounted for most of the cases when compared with other histological types. More than 80% of the patients were diagnosed at stages III and IV.

### 3.2. Variation Status

Overall, 77 variants were observed in 72 individuals after filtration. These variants were classified into the following categories: 30 as PVs, 10 as likely pathogenic variants (LPVs), and 37 as VUSs. Based on the type of variation, 36 were missense, 21 were frameshift, 9 were nonsense, and 6 were splice variants. In addition, 5 large fragment deletions were found in 6 patients. 8 of the 77 variants could not be retrieved in related public databases and were considered to be novel (Table 2).

### 3.3. PVs and LPVs

Sequencing results of the 18-gene panel showed that 43 (36.44%) of the 118 patients carried at least 1 PV/LPV (Table S2). Among these, *BRCA1* PVs/LPVs were detected in 31 (26.27%) patients and 5 (4.24%) patients carried PVs/LPVs of *BRCA2*. The remaining 7 (5.93%) patients carried PVs/LPVs in non-*BRCA1/2* genes (*ATM*, *BRIP1*, *CHEK2*, *MSH6*, *TP53*, and *STK11*). Half of the PVs/LPVs were found to be frameshift variants.

### 3.4. VUSs

In this study, 37 VUSs were identified in 32 (27.12%) patients (Table S3). 26 of 32 patients carried VUS in non-*BRCA1/2* genes while 8 (25%) of 32 patients carried VUS in *BRCA1/2* genes (2 patients carried both *BRCA1/2* and non-*BRCA* variants). It was found that at least 1 VUS was identified in other 16 genes, except in *PTEN* and *STK11*. In addition, 8 patients carried 2 VUSs, and 1 patient carried 3 VUSs. Unlike PVs/LPVs, almost all VUSs (33/37) were missense variants. Interestingly, three unrelated patients (P24, P81, and P86) carried the same variants of *MUTYH* c.74G>A p.(Gly25Asp) and *MUTYH* c.53C>T p.(Pro18Leu).

## 4. Discussion

Although there have been similar studies abroad, there exist few studies on multigene panel testing of OC patients in the Chinese population. In this study, 118 patients were screened for the presence of sequence variants in 18 HOC-associated genes. PVs/LPVs variants were identified in 43 (36.44%) patients, and most of them were identified in *BRCA1/2* (30.51%). We also detected 37 VUSs in 32 individuals, and a majority of these were identified in *ATM*, *PMS2*, *MUTYH*, and *CDH1*. In addition, 8 novel variants were reported.

**Figure 1:**
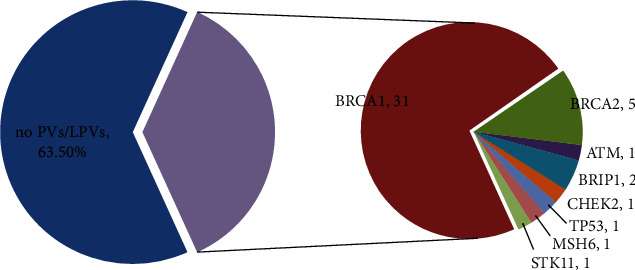
Distribution of PVs/LPVs in this study. 43 out of 118 patients were identified with pathogenic/likely pathogenic variants. PVs: pathogenic variants; LPVs: likely pathogenic variants.

Compared with other studies, our *BRCA1/2* gene PVs/LPVs rate (30.51%) was higher than that of other races (ranging from 13.3% to 18%) [13–15]. However, this finding was in concordance with studies in the Chinese population. This rate was 23.07% in Shao's study [16] and 28.5% in Wu's study [17]. This may be related to the genetic background of different races. In addition, 7 (5.93%) patients carried non-*BRCA1/2* PVs/LPVs, which were found in *ATM*, *CHEK2*, *BRIP1*, *MSH6*, *TP53*, and *STK11* (Figure 1). Among them, 2 patients showed PVs/LPVs in *BRIP1*. *BRIP1* is a member of the Fanconi anemia pathway. It participates in DNA interstrand cross-link repair [18, 19]. Many studies have shown that *BRIP1* is associated with an increased risk for OC [20, 21]. It is estimated that the cumulative lifetime risk of developing OC by 80 years of age in *BRIP1* PVs/LPVs carriers is 5.8% [20]. A novel *BRIP1* c.168_169insA p.(Leu57Thrfs∗12) was identified in an OC patient diagnosed at age 47 (P55) (Table 2). This variation causes the gene encoding protein to terminate prematurely at position 68, causing its polypeptide chain to be truncated, while the normal gene can encode 1249 amino acids.

One of the patients in the study (P18) carried 2 novel VUSs simultaneously and was diagnosed with fallopian tube cancer at age 54 (Table 2). The variants were located at the *ATM* and *NBN*, respectively. The *ATM* protein kinase is best known for its role as a chief mobilizer of the cellular response to this DNA lesion, and biallelic pathogenic *ATM* variants cause Ataxia Telangiectasia [22]. *ATM* c.7084G>C p.(Glu2362Gln) leads to the variation of glutamate at position 2362 to glutamine. The replacement of an acidic amino acid with a neutral amino acid may lead to a change in the structure of the *ATM* protein and influence its function.

The other novel VUS identified in P18 was *NBN* c.1037T>C p.(Val346Ala), resulting in the variation of valine at position 346 to alanine. Existing scientific research is still unable to clarify its relationship with the risk of OC. The *NBN* gene is located on chromosome 8 and is responsible for producing the protein nibrin. It regulates cellular reactions to DNA breakdown and maintenance of chromosomal stability [23].

In our study, we selected 18 genes to build a panel in order to investigate the variation frequency of HOC susceptibility genes. Among these genes, *BRCA1*, *BRCA2*, *PALB2*, *BRIP1*, *MLH1*, *MSH2*, *MSH6*, and *RAD51C* have been proved to increase the risk of OC [11]. However, the relationship between *ATM, CHEK2*, *TP53*, *PTEN, CDH1*, *MUTYH*, *NBN*, *PMS2*, and the risk of OC is still controversial. *STK11* has only been confirmed to be related to nonepithelial ovarian tumors. Our study incorporates both moderate- and high-penetrance genes and provides more updated data on the genetic etiology of epithelial OC in the Chinese population.

NCCN guidelines have confirmed that genetic testing should be offered to individuals with any blood relative with a known PV/LPV in a cancer susceptibility gene. For the PVs/LPVs we detected in this study, if the family members of these patients have also detected *BRCA*/*BRIP1*/*MSH6* PVs/LPVs, risk-reducing salpingo-oophorectomy (RRSO) can be performed in advance [11]. For ATM PVs/LPVs carriers, there is currently insufficient evidence to recommend RRSO. Germline *STK11* PVs/LPVs are associated with Peutz-Jeghers syndrome, and TP53 are associated with Li-Fraumeni Syndrome. There are currently no recommendations to reduce the risk of OC for carriers of these two gene PVs/LPVs. The management of these patients and their family members still needs further study.

There are several limitations to our study. First, we need a much larger study cohort to detect more variants in the Chinese population. Second, the panel we designed in this study included 18 genes that may not cover all high-penetrance genes associated with HOC. Third, further research is needed to clarify the correlation of sequence variants with clinical features and prognosis.

Our study enlarged the spectrum of HOC-associated gene sequence variants in the Chinese population and also proved the necessity of multigene testing in epithelial OC patients. The identification of patients with HOC allows the patient to benefit from personalized treatment. It also allows family members to undergo cascade testing where identification of unaffected carriers can facilitate early detection, risk reduction, or prevention of OC and ultimately improve long-term outcomes. The screening and management of high-risk women who carry PVs/LPVs, especially non-*BRCA* variants, need further research.

## Figures and Tables

**Table 1:** Clinical characteristics of 118 patients included in the present study (all histology results are jointly reported by two experienced pathologists).

Characteristics	Total (*n* = 118)	Percent (%)
Age at diagnosis		
30-39	9	7.63%
40-49	34	28.81%
50-59	53	44.92%
60-69	16	13.56%
70-79	6	5.08%
Stage		
I	12	10.17%
II	7	5.93%
III	80	67.80%
IV	19	16.10%
Histology		
Serous	103	87.29%
Endometrioid	4	3.39%
Mucinous	6	5.08%
Clear cell	5	4.24%
Self, in addition to OC		
Breast cancer	4	3.39%
No breast cancer	1	0.85%
Family history		
Breast cancer	2	1.69%
Ovarian cancer	5	4.24%
Pancreatic cancer	4	3.39%
Uterine cancer	1	0.85%
Colon cancer	1	0.85%

**Table 2:** Novel variants identified in participants.

Gene	Variant	Protein change	Function change	Cadd	Dann	SIFT score pred	POLYPHEN score pred	Mutationtaster score pred	Class	Patient	Diagnosed age
BRCA1	c.2901delT	p.Pro968Glnfs∗32	Frameshift	—	—	—	—	—	4	P5	50
BRCA1	c.5439delT	p.Asp1813Glufs∗21	Frameshift	—	—	—	—	—	4	P58	54
BRCA1	c.2971_2975del AAAAC	p.Lys991∗	Frameshift	—	—	—	—	—	4	P85	36
BRCA1	c.2483del	p.Gly828fs	Frameshift	—	—	—	—	—	5	P107	49
BRCA2	c.3861delT	p.Asn1287LysfsX6	Frameshift	—	—	—	—		4	P4	54
ATM	c.7084G>C	p.Glu2362Gln	Missense	3.5636	0.998	D	D	D	3	P18	54
BRIP1	c.168_169insA	p.Leu57Thrfs∗12	Frameshift	—	—	—	—	—	4	P55	47
NBN	c.1037T>C	p.Val346Ala	Missense	-0.0005	0.643	T	B	N	3	P18	54

Variants are named according to Human Genome Variation Society (HGVS) nomenclature. SIFT score pred: D: damaging; T: tolerated; POLYPHEN score pred: B: benign; P: possibly damaging; D: probably damaging; MutationTaster score pred: D: disease causing; N: polymorphism.

## Data Availability

The datasets used and/or analysed during the current study are available from the corresponding authors on reasonable request.

## Supplementary Materials

Supplementary Materials Table S1: overview of the 18 genes investigated in this study. Table S2: PVs/LPVs identified in this study. Table S3: variants of uncertain significance identified in this study.
