# Supplementary material for: Identifying Sequence Variants of 18 Hereditary Ovarian Cancer-Associated Genes in Chinese Epithelial Ovarian Cancer Patients
Source: Biomed Res Int. 2021 Jul 24;2021:5579543. doi: 10.1155/2021/5579543 (PMC8328723; doi:10.1155/2021/5579543)
Supplement: Supplementary Materials — Table S1: overview of the 18 genes investigated in this study. Table S2: PVs/LPVs identified in this study. Table S3: variants of uncertain significance identified in this study. [file 5579543.f1.zip › Table S1.doc]

**Table S1**. Overview of the 18 genes investigated in this study

| Name | Description | Location | Reference sequence | Numbers of exons |
| --- | --- | --- | --- | --- |
| BRCA1 | BRCA1 DNA repair associated | Chromosome 17, NC_000017.11 (43044295..43125364, complement) | NM_007294 | 23 |
| BRCA2 | BRCA2 DNA repair associated | Chromosome 13, NC_000013.11 (32315508..32400268) | NM_000059 | 27 |
| CHEK2 | Checkpoint kinase 2 | Chromosome 22, NC_000022.11 (28687743..28741866, complement) | NM_007194 | 16 |
| PALB2 | Partner and localizer of BRCA2 | Chromosome 16, NC_000016.10 (23603162..23641337, complement) | NM_024675 | 13 |
| BRIP1 | BRCA1 interacting protein C-terminal helicase 1 | Chromosome 17, NC_000017.11 (61679139..61864120, complement) | NM_032043 | 20 |
| TP53 | Tumor protein p53 | Chromosome 17, NC_000017.11 (7668402..7687550, complement) | NM_000546 | 11 |
| PTEN | Phosphatase and tensin homolog | Chromosome 10, NC_000010.11 (87863625..87971930) | NM_00314 | 9 |
| STK11 | Serine/threonine kinase 11 | Chromosome 19, NC_000019.10 (1205778..1228431) | NM_000455 | 10 |
| CDH1 | Cadherin 1 | Chromosome 16, NC_000016.10 (68737292..68835537) | NM_004360 | 16 |
| ATM | ATM serine/threonine kinase | Chromosome 11, NC_000011.10 (108222484..108369102) | NM_000051 | 63 |
| BARD1 | BRCA1 associated RING domain 1 | Chromosome 2, NC_000002.12 (214725645..214809683, complement) | NM_000465 | 13 |
| MLH1 | MutL homolog 1 | Chromosome 3, NC_000003.12 (36993350..37050846) | NM_000249 | 19 |
| MSH2 | MutS homolog 2 | Chromosome 2, NC_000002.12 (47403067..47634501) | NM_000251 | 16 |
| MSH6 | MutS homolog 6 | Chromosome 2, NC_000002.12 (47783082..47806954) | NM_000179 | 10 |
| MUTYH | MutY DNA glycosylase | Chromosome 1, NC_000001.11 (45329242..45340470, complement) | NM_001128425 | 22 |
| NBN | Nibrin | Chromosome 8, NC_000008.11 (89933331..89984724, complement) | NM_002485 | 16 |
| PMS2 | PMS1 homolog 2, mismatch repair system component | Chromosome 7, NC_000007.14 (5970925..6009106, complement) | NM_000535 | 15 |
| RAD51C | RAD51 paralog C | Chromosome 17, NC_000017.11 (58692140..58735611) | NM_058216 | 9 |
