# Supplementary material for: Identifying Sequence Variants of 18 Hereditary Ovarian Cancer-Associated Genes in Chinese Epithelial Ovarian Cancer Patients
Source: Biomed Res Int. 2021 Jul 24;2021:5579543. doi: 10.1155/2021/5579543 (PMC8328723; doi:10.1155/2021/5579543)
Supplement: Supplementary Materials — Table S1: overview of the 18 genes investigated in this study. Table S2: PVs/LPVs identified in this study. Table S3: variants of uncertain significance identified in this study. [file 5579543.f1.zip › Table S2.doc]

**Table S2**. Deleterious variants identified in this study

| Gene | Variant | Protein change | Function Change | Databases | | | | Cl. | Patient | Age |
| --- | --- | --- | --- | --- | --- | --- | --- | --- | --- | --- |
|  |  |  |  | Population databases | HGMD | dbSNP | Clinvar |  |  |  |
| BRCA1 | c.4485-2A>C | - | Splice | 1000g_ALL:-;ExAc:-;gnomAD:- | - | rs80358054 | VCV000567954.2; LP | 4 | P1 | 46 |
| BRCA1 | c.2719G>T | p.Glu907Ter | Nonsense | 1000g_ALL:-;ExAc:-;gnomAD:- | CM176578 | rs876658593 | VCV000230485.2; P | 4 | P2 | 40 |
| BRCA1 | c.2901delT | p.Pro968Glnfs*32 | Frameshift | 1000g_ALL:-;ExAc:-;gnomAD:- | - | - | - | 4 | P5 | 50 |
| BRCA1 | c.5470_5477 delATTGGGCA | p.Ile1824AspfsX3 | Frameshift | 1000g_ALL:-;ExAc:-;gnomAD:- | CD043938 | rs80357973 | VCV000055591.6; P | 5 | P6 | 48 |
| BRCA1 | c.2920_2921 delTT | p.Leu974ThrfsX17 | Frameshift | 1000g_ALL:-;ExAc:-;gnomAD:- | CD972056 | rs80357611 | VCV000054718.1; P | 5 | P7 | 45 |
| BRCA1 | c.3770_3771delAG | p.Glu1257GlyfsX9 | Frameshift | 1000g_ALL:-;ExAc:0%;gnomAD:0% | CD961840 | rs80357579 | VCV000037546.7; P | 5 | P9 | 55 |
| BRCA1 | c.81-2A>G | - | Splice | 1000g_ALL:-;ExAc:-;gnomAD:- | CS109760 | rs397509326 | VCV000055716.5; LP | 5 | P14 | 51 |
| BRCA1 | c.4573C>T | p.Gln1525Ter | Nonsense | 1000g_ALL:-;ExAc:-;gnomAD:- | CM1413407 | rs886040237 | VCV000266484.1; P | 5 | P38 | 50 |
| BRCA1 | c.3700_3704del GTAAA | p.Val1234Glnfs X8 | Frameshift | 1000g_ALL:-;ExAc:-;gnomAD:- | CD951619 | rs80357609 | VCV000037542.13 P | 5 | P49 | 55 |
| BRCA1 | c.981_982delAT | p.Cys328* | Frameshift | 1000g_ALL:-;ExAc:-;gnomAD:- | CD961826 | rs80357772 | VCV000055772.7; P | 5 | P52;P84 | 50;45 |
| BRCA1 | c.5439delT | p.Asp1813Glufs*21 | Frameshift | 1000g_ALL:-;ExAc:-;gnomAD:- | - | - | - | 4 | P58 | 54 |
| BRCA1 | c.5282T>C | p.Phe1761Ser | Missense | 1000g_ALL:-;ExAc:-;gnomAD:- | CM165047 | rs80356905 | VCV000055505.4:CIOP | 4 | P68 | 72 |
| BRCA1 | c.1252G>T | p.Glu418Ter | Nonsense | 1000g_ALL:-;ExAc:-;gnomAD:- | CM122311 | rs80357083 | VCV000054176.3; P | 5 | P69;p80 | 39;34 |
| BRCA1 | c.2799delT | p.Gln934Argfs*66 | Frameshift | 1000g_ALL:-;ExAc:-;gnomAD:- | - | rs80357998 | VCV000125590.2; P | 5 | P72 | 51 |
| BRCA1 | c.3359_3363 delTTAAT | p.Val1120Aspfs*11 | Frameshift | 1000g_ALL:-;ExAc:-;gnomAD:- | CD065674 | rs397509060 | VCV000054857.2; P | 5 | P78 | 58 |
| BRCA1 | c.2702_2703 delTT | p.Phe901* | Frameshift | 1000g_ALL:-;ExAc:-;gnomAD:- | CD011117 | rs80357899 | VCV000054653.1; P | 5 | P79 | 63 |
| BRCA1 | c.2971_2975del AAAAC | p.Lys991* | Frameshift | 1000g_ALL:-；ExAc:-；gnomAD:- | - | - | - | 4 | P85 | 36 |
| BRCA1 | c.212G>A | p.Arg71Lys | Missense | 1000g_ALL:-ExAc:-;  gnomAD:- | CM021504 | rs80356913 | VCV000054471.3; P | 5 | P97 | 53 |
| BRCA1 | c.220C>T | p.Gln74* | Nonsense | 1000g_ALL:-;ExAc:-;gnomAD:- | CM970167 | rs80357234 | VCV000054499.5; Pathogenic | 5 | P99 | 49 |

(Continues)

Table S2. Deleterious variants identified in this study(Continued)

| Gene | Variant | Protein change | Function Change | Databases | | | | Cl. | Patient | Age |
| --- | --- | --- | --- | --- | --- | --- | --- | --- | --- | --- |
|  |  |  |  | Population databases | HGMD | dbSNP | Clinvar |  |  |  |
| BRCA1 | c.2405_2406del | p.Val802fs | Frameshift | 1000g_ALL:-ExAc:0%;gnomAD:0% | CD011116 | rs80357706 | VCV000054558.5; P | 5 | P102 | 40 |
| BRCA1 | c.2483del | p.Gly828fs | Frameshift | 1000g_ALL:-;ExAc:-;gnomAD:- | - | - | - | 5 | P107 | 49 |
| BRCA1 | c.4185+1G>A | - | Splice | 1000g_ALL:-;ExAc:-;  gnomAD:- | CS1411526 | rs80358076 | VCV000186395.2; P | 5 | P111 | 66 |
| BRCA1 | c.4013del | p.Lys1338fs | Frameshift | 1000g_ALL:-;ExAc:-;  gnomAD:- | CD1715219 | rs886038029 | VCV000254449.1; P | 5 | P114 | 42 |
| BRCA1 | c.1465G>T | p.Glu489* | Nonsense | 1000g_ALL:-;ExAc:-;  gnomAD:- | CM023341 | rs80357167 | VCV000054262.2; P | 5 | P118 | 47 |
| BRCA2 | c.3861delT | p.Asn1287LysfsX6 | Frameshift | 1000g_ALL:-;ExAc:-;  gnomAD:- | - | - | - | 4 | P3 | 58 |
| BRCA2 | c.2306delT | p.Ile770Phefs*2 | Frameshift | 1000g_ALL:-；ExAc:-;  gnomAD:- | - | - | - | 4 | P4 | 54 |
| BRCA2 | c.2095C>T | p.Gln699Ter | Nonsense | 1000g_ALL:-;ExAc:-;  gnomAD:- | CM134881 | rs878853559 | VCV000236835.10 P | 5 | P42 | 53 |
| BRCA2 | c.2257delT | p.Gln754Asnfs*18 | Frameshift | 1000g_ALL:-;ExAc:-;  gnomAD:- | CD042856 | rs397507621 | VCV000051261.1; P | 5 | P70 | 50 |
| BRCA2 | c.1293_1294d elAG | p. Asn433Glnfs*18 | Frameshift | 1000g_ALL:-;ExAc:-;  gnomAD:- | CD152264 | rs80359276 | VCV000037736.9; P | 5 | P75 | 53 |
| ATM | c.5869_5870delTA | p.Tyr1957Cysfs*7 | Frameshift | 1000g_ALL:-；ExAc:-;  gnomAD:0.00% | CD1715158 | rs1060501657 | VCV000407654.2; P | 5 | P76 | 53 |
| BRIP1 | c.2392C>T | p.Arg798Ter | Nonsense | 1000g_ALL:-;ExAc:0.02%；gnomAD:0.02% | CM053140 | rs137852986 | VCV000004738.14 CIOP | 5 | P47 | 50 |
| BRIP1 | c.168_169insA | p.Leu57Thrfs*12 | Frameshift | 1000g_ALL:-;ExAc:-;  gnomAD:- | - | - | - | 4 | P55 | 47 |
| CHEK2 | c.283C>T | p.Arg95* | Nonsense | 1000g_ALL:-;ExAc:0%;gnomAD：0% | CM110734 | rs587781269 | VCV000140772.9; P | 5 | P92 | 68 |
| MSH6 | c.3202 C>T | p.Arg1068Ter | Nonsense | 1000g_ALL:-；ExAc:-;  gnomAD:- | CM021637 | rs63749843 | VCV000089352.10 P | 5 | P13 | 51 |
| TP53 | c.818G>A | p.Arg273His | Missense | 1000g_ALL：0.02%；ExAc：0%；gnomAD：0% | CM920677 | rs28934576 | VCV000012366.8; P | 5 | P96 | 44 |
| BRCA1 | EX8 DEL | - | - | - | - | - | - | 5 | P8 | 56 |
| BRCA1 | EX1_2 DEL | - | - | - | - | - | - | 5 | P10;P11 | 55;52 |
| BRCA1 | EX20 DEL | - | - | - | - | - | - | 5 | P12 | 43 |
| BRCA1 | EX13_16 DEL | - | - | - | - | - | - | 5 | P34 | 50 |
| STK11 | EX1_9 DEL | - | - | - | - | - | - | 4 | P37 | 61 |

Variants are named according to Human Genome Variation Society (HGVS) nomenclature. Cl.: class (4: likely pathogenic; 5: pathogenic); P: pathogenic; LP: likely pathogenic; CIOP= conflicting interpretations of pathogenicity; Age: Diagnosed age
