# Supplementary material for: Identifying Sequence Variants of 18 Hereditary Ovarian Cancer-Associated Genes in Chinese Epithelial Ovarian Cancer Patients
Source: Biomed Res Int. 2021 Jul 24;2021:5579543. doi: 10.1155/2021/5579543 (PMC8328723; doi:10.1155/2021/5579543)
Supplement: Supplementary Materials — Table S1: overview of the 18 genes investigated in this study. Table S2: PVs/LPVs identified in this study. Table S3: variants of uncertain significance identified in this study. [file 5579543.f1.zip › Table S3.doc]

Table S3. Variants of uncertain significance identified in this study

| Gene | Variant | Protein change | Function Change | Databases | | | | Patient | Age |
| --- | --- | --- | --- | --- | --- | --- | --- | --- | --- |
|  |  |  |  | Population database | HGMD | dbSNP | Clinvar |  |  |
| BRCA1 | c.3541G>A | p.Val1181Ile | Missense | 1000g_ALL:0.04%;ExAc:0.01%;gnomAD:0.01% | - | rs56336919 | VCV000054911.8; B | P17 | 63 |
| BRCA1 | c.3448C>T | p.Pro1150Ser | Missense | 1000g_ALL:0.08%;ExAc:0.01%;gnomAD:0.01% | CM041694 | rs80357272 | VCV000054887.9; B | P61 | 61 |
| BRCA1 | c.1966A>T | p.Asn656Tyr | Missense | 1000g_ALL:-;ExAc:-;  gnomAD:- | CM1514828 | rs786203455 | VCV000559977.1; VUS | P118 | 47 |
| BRCA2 | c.8350 C>T | p.Arg2784Trp | Missense | 1000g_ALL:-;ExAc:0.00%;  gnomAD:0.00% | CM1612912 | rs80359075 | VCV000038155.11 CIOP | P19 | 46 |
| BRCA2 | c.7522G>A | p.Gly2508Ser | Missense | 1000g_ALL:-;ExAc：0.01%；gnomAD：0.02% | CM097396 | rs80358978 | VCV000052347.8; CIOP | P91 | 54 |
| BRCA2 | c.9116C>T | p.Pro3039Leu | Missense | 1000g_ALL:-;ExAc:0.01%;  gnomAD:0.01% | CM1619570 | rs80359167 | VCV000052753.6; CIOP | P93 | 58 |
| BRCA2 | c.112G>A | p.Glu38Lys | Missense | 1000g_ALL:-;ExAc:-;  gnomAD:- | - | rs1555280344 | VCV000479376.6; VUS | P94 | 51 |
| BRCA2 | c.8009C>G | p.Ser2670Trp | Missense | 1000g_ALL:-;ExAc:-;  gnomAD:- | CM179902 | rs80359035 | VCV000489785.4; VUS | P113 | 65 |
| ATM | c.7084G>C | p.Glu2362Gln | Missense | 1000g_ALL:-;ExAc:-;  gnomAD:0.00% | - | - | - | P18 | 54 |
| ATM | c.4949 A>G | p.Asn1650Ser | Missense | 1000g_ALL:0.24%;ExAc:0.05%;gnomAD:0.06% | - | rs55870064 | VCV000127395.9; B/LB | P23 | 67 |
| ATM | c.7896C>G | p.Asn2632Lys | Missense | 1000g_ALL:-;ExAc:-;  gnomAD:- | - | - | VCV000941254.1; VUS | P63 | 43 |
| ATM | c.1483A>G | p.Ile495Val | Missense | 1000g_ALL:-;ExAc:-;  gnomAD:- | - | rs786201969 | VCV000185158.3; VUS | P98 | 46 |
| ATM | c.8797A>G | p.Lys2933Glu | Missense | 1000g_ALL:-;ExAc:0.00%;  gnomAD:0.00% | - | rs587779875 | VCV000127464.6; VUS | P103 | 41 |
| BARD1 | c.26_40delACCGGCAGCCGAGGA | p.Asn9_Arg13del | Frameshift | 1000g_ALL:-;ExAc:0.01%;  gnomAD:0.00% | - | rs587781979 | VCV000141745.8; VUS | P54 | 55 |
| BRIP1 | c.2440C>T | p.Arg814Cys | Missense | 1000g_ALL:0.10%;ExAc:0.05%;gnomAD:0.05% | CM161719 | rs201869624 | VCV000133755.7; CIOP | P25 | 72 |
| CDH1 | c.604 G>A | p.Val202Ile | Missense | 1000g_ALL:0.20%;ExAc:0.06%;gnomAD:0.05% | CM173860 | rs546716073 | VCV000133853.6; B | P16 | 53 |
| CDH1 | c.2494G>A | p.Val832Met | Missense | 1000g_ALL:0.04%;ExAc:0.02%;gnomAD:0.02% | CM023731 | rs35572355 | VCV000012246.9;B | P40 | 39 |
| CDH1 | c.1296C>G | p.Asn432Lys | Missense | 1000g_ALL:-;ExAc:0.00%;  gnomAD:0.00% | - | rs187862045 | VCV000186937.6; VUS | P51 | 54 |
| CHEK2 | c.1438G>A | p.Ala480Thr | Missense | 1000g_ALL:-;ExAc:0.00%;  gnomAD:0.00% | - | rs773600515 | VCV000230405.5; VUS | P97 | 53 |

(Continues)

Table S3. Variants of uncertain significance identified in this study (Continued)

| Gene | Variant | Protein change | Function Change | Databases | | | | Patient | Age |
| --- | --- | --- | --- | --- | --- | --- | --- | --- | --- |
|  |  |  |  | Population databases | HGMD | dbSNP | Clinvar |  |  |
| MLH1 | c.319 A>G | p.Ile107Val | Missense | 1000g_ALL:0.02%;ExAc:0.00%;gnomAD:0.00% | - | rs572906317 | VCV000188068.6; VUS | P21 | 58 |
| MSH2 | c.2344A>G | p.Thr782Ala | Missense | 1000g_ALL:-;ExAc:-;  gnomAD:- | - | rs35784190 | - | P41 | 35 |
| MSH2 | c.2197G>A | p.Ala733Thr | Missense | 1000g_ALL:-;ExAc:0.00%;  gnomAD:0.00% | CM174245 | rs772662439 | VCV000216350.8; VUS | P51 | 54 |
| MSH6 | c.1051C>T | p.His501Tyr | Missense | 1000g_ALL:-;ExAc:-;  gnomAD:- | - | rs587779911 | VCV000127553.2; VUS | P44 | 69 |
| MSH6 | c.1063G>A | p.Gly355Ser | Missense | 1000g_ALL:-;ExAc:0.01%;  gnomAD:0.01% | CM1511870 | rs587778531 | VCV000134851.5; CIOP | P107 | 49 |
| MUTYH | c.74G>A | p.Gly25Asp | Missense | 1000g_ALL:0.18%;ExAc:0.11%;gnomAD:0.11% | CM083730 | rs75321043 | VCV000041763.11; CIOP | P24;P81;P86 | 67;52;53 |
| MUTYH | c.53C>T | p.Pro18Leu | Missense | 1000g_ALL:0.18%;ExAc:0.11%;gnomAD:0.11% | CM083727 | rs79777494 | VCV000041759.12; CIOP | P24;P81;P86 | 67;52;53 |
| MUTYH | c.572G>A | p.Arg191Gln | Missense | 1000g_ALL:-;ExAc:0.00%;  gnomAD:0.01% | - | rs369677603 | VCV000127846.7; CIOP | P94 | 51 |
| NBN | c.37+6 G>A | - | Splice | 1000g_ALL:0.02%;ExAc:0.01%;gnomAD:0.01% | - | rs540868733 | VCV000411792.3; CIOP | P15;P90 | 53;37 |
| NBN | c.1037T>C | p.Val346Ala | Missense | 1000g_ALL:-;ExAc:-;  gnomAD:- | - | - | - | P18 | 54 |
| PALB2 | c.319T>A | p.Phe107Ile | Missense | 1000g_ALL:-;ExAc:0.00%;  gnomAD:0.00% | - | rs752251774 | VCV000460979.2; VUS | P65 | 42 |
| PALB2 | c.290C>A | p.Ser97Tyr | Missense | 1000g_ALL:-;ExAc:0.00%;  gnomAD:0.00% | CM175013 | rs776902295 | VCV000460968.3; VUS | P65 | 42 |
| PMS2 | c.166C>G | p.Leu56Val | Missense | 1000g_ALL:-;ExAc:0.00%;  gnomAD:0.00% | - | rs371011390 | VCV000127765.6; CIOP | P56 | 54 |
| PMS2 | c.1145-10G>A | - | Splice | 1000g_ALL:0.02%;ExAc:0.00%;gnomAD:0.01% | - | rs533551639 | VCV000237877.3; CIOP | P81 | 52 |
| PMS2 | c.1318C>G | p.Pro440Ala | Missense | 1000g_ALL:-;ExAc:0.00%;  gnomAD:0.00% | - | rs762894051 | VCV000233783.4; VUS | P93 | 58 |
| PMS2 | c.451C>T | p.Arg151Cys | Missense | 1000g_ALL:-;ExAc:0.00%;  gnomAD:0.00% | - | rs758561884 | VCV000231932.5; VUS | P97 | 53 |
| RAD51C | c.571+5G>A | - | Splice | 1000g_ALL:0.02%;ExAc:0.00%;gnomAD:0.00% | - | rs145779113 | VCV000216805.6; VUS | P101 | 65 |
| TP53 | c.868 C>T | p.Arg290Cys | Missense | 1000g_ALL:-;ExAc:0.00%;  gnomAD:0.00% | CM1712986 | rs770374782 | VCV000216472.7; CIOP | P22 | 42 |

Variants are named according to Human Genome Variation Society (HGVS) nomenclature. CIOP: Conflicting interpretations of pathogenicity; B: benign; LB: likely benign; VUS: variants of uncertain significance;CIOP= conflicting interpretations of pathogenicity; Age: Diagnosed age
